# Cloud Point Extraction of Parabens Using Non-Ionic Surfactant with Cylodextrin Functionalized Ionic Liquid as a Modifier

**DOI:** 10.3390/ijms141224531

**Published:** 2013-12-17

**Authors:** Md Saleh Noorashikin, Muggundha Raoov, Sharifah Mohamad, Mhd Radzi Abas

**Affiliations:** 1 Environmental Research Group, Department of Chemistry, Faculty of Science, University of Malaya, Kuala Lumpur 50603, Malaysia; E-Mail: radzi@um.edu.my (M.R.A); 2 Department of Chemical Sciences, Faculty of Science and Technology, Universiti Malaysia Terengganu, Kuala Terengganu 21030, Malaysia; E-Mail: noorashikin@siswa.um.edu.my; 3 Advanced Medical & Dental Institute, University of Science Malaysia, No 1–8 (Lot 8), Persiaran Seksyen 4/1, Bandar Putra Bertam, Kepala Batas, Pulau Pinang 13200, Malaysia; E-Mail: muggundha_raoov@hotmail.com

**Keywords:** cloud point extraction, non-ionic surfactant, β-cyclodextrin-ionic liquid, paraben, water sample

## Abstract

A cloud point extraction (CPE) process using non-ionic surfactant (DC193C) to extract selected paraben compounds from water samples was investigated using reversed phase high performance liquid chromatography (RP-HPLC). The CPE process with the presence of β-cyclodextrin (βCD) functionalized ionic liquid as a modifier (CPE-DC193C-βCD-IL) is a new extraction technique that has been applied on the optimization of parameters, *i.e.*, pH, βCD-IL concentration and phase volume ratio. This CPE-DC193C-βCD-IL method is facilitated at 30 °C, showing great losses of water content in the surfactant-rich phase, resulting in a high pre-concentration factor and high distribution coefficient. The developed method CPE-DC193C-βCD-IL did show enhanced properties compared to the CPE method without the modifier (CPE-DC193C). The developed method of CPE-DC193C-βCD-IL gives an excellent performance on the detection of parabens from water samples with the limit of detection falling in the range of 0.013–0.038 μg mL^−1^. Finally, the inclusion complex formation, hydrogen bonding, and π–π interaction between the βCD-IL, benzyl paraben (ArP), and DC 193C were proven using ^1^H NMR and 2D NOESY spectroscopy.

## 1. Introduction

Parabens are effective preservatives in many types of formulas. They can be found in shampoos, commercial moisturizers, shaving gels, personal lubricants, topical pharmaceuticals, spray tanning solutions, make-up and toothpastes. They are also used as food additives. These compounds are considered as endocrine-disrupting chemicals (ECDs) because of their endocrine activity [1–3] and the fact that they have been detected in human tissues including breast tumors [4]. Therefore, developing a reliable method for determining parabens in our environment should be a major concern. A number of studies were conducted to investigate persistence and partitioning of parabens in water samples. This is because some residues may get into the water after being discharged from industrial effluents or pharmaceuticals and cosmetic products. The Environmental Protection Agency (EPA) has certified the liquid–liquid extraction (LLE) as a method for extraction of organic pollutants from environmental samples. However, the LLE method requires a large amount of organic solvents. For the purpose of eliminating or at least minimizing the use of organic solvents, many sample pretreatment methods have been developed. Among them, the most common pretreatment methods are supercritical fluid extraction (SFE) [5], solid-phase extraction (SPE) [6], solid-phase microextraction (SPME) [6] and liquid-phase microextraction (LPME) [7].

The application of the cloud point extraction in aqueous media for the analytical determination of trace organic analytes has aroused growing attention over the past a few years [8–10]. Cloud point has been demonstrated where these techniques are able to extract and pre-concentrate a wide range of organic compounds from the aqueous phase [11–13]. The cloud point extraction techniques result in fast extraction, high preconcentration factor, and the removal of the need to use toxic and environmentally unfriendly organic solvents [14–16] that make it advantageous compared to other techniques.

Cyclodextrin (CD) or cyclomaltoheptases are well-known series of macro-cyclic oligosaccharides resulting from the degradation of starch by bacterial enzymes. Generally, CDs are composed of 6, 7, or 8 D-glucose units connected by α-1,4-glucosidic linkages which are categorized as αCD, βCD and γCD, respectively. Every D-glucose unit consists of three free hydroxyl unit groups, which differ in their reactivity and functions [17]. The entire primary hydroxyl group at the 6-positions of the D-glucose units is on the other side of the ring, while the entire secondary hydroxyl group at the 2 and 3-positions is on one side of the torus. The most notable feature of βCD is its ability to form solid inclusion compounds (host–guest complexes) with a wide range of solid, liquid, and gaseous compounds by molecular complexation [18–20] and through various kinds of interaction (van der Waals force, hydrophobic interaction, electrostatic affinity, dipole–dipole interaction, and hydrogen bonding) [21]. Due to specific properties of CD materials, it provides a wide range of research and application [22]. Examples of applications include chemical separations [23], adsorbents [24,25], food processing [26], and pharmaceutical excipients [27].

Ionic liquid (IL), is a kind of salt in which the ions are poorly coordinated. Consequently, these compounds are liquid below 100 °C or even at room temperature (RTILs) [28]. They have unique properties such as non-volatility, non-flammability, low viscosity, chemical and electrochemical stability [29], and remain in the liquid state over a wide temperature range. ILs can also be designed to be environmentally benign, with large potential benefit for sustainable chemistry [30]. ILs are considered as templating solvents in some synthesis due to their ability to self-assemble in different domains (polar and non polar) and these properties of ILs have been transferred to the development of supramolecular materials [31]. Owing to the properties of βCD and ILs, the functionalization of βCD with IL has fostered our interest in preparing a new generation of material that may demonstrate some interesting phenomena in extraction studies. To the best of our knowledge, the CD functionalized ionic liquid materials were widely used as chiral selectors in capillary electrophoresis [32] and stationary phase in HPLC [33–35], while CPE extraction is still in its infancy. Hence, this study will serve as a preliminary work for the extraction of parabens through the CPE method using βCD functionalized with ionic liquid as a modifier.

In this study, βCD functionalized IL (βCD-IL) was used as a modifier in the CPE system in order to determine parabens from water in a simple, fast and efficient method, using experiments that are low cost and contribute to green technology. The obtained results were compared with the CPE system without βCD-IL as modifier (CPE-DC193C). The developed methods will be tested in the extraction of parabens from real water. Finally, the inclusion complex of βCD-IL with DC193C and benzyl paraben has been investigated in order to propose the extraction mechanism.

## 2. Results and Discussion

### 2.1. Effect of pH on Extraction Recoveries of Parabens Extraction

**Figure 1.**
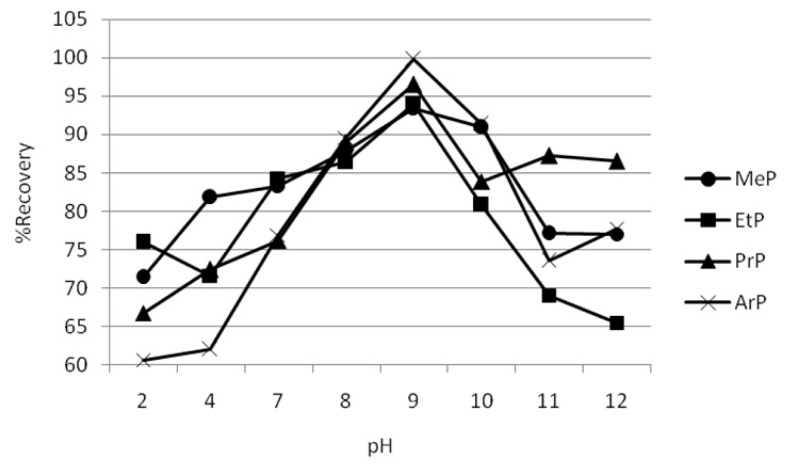
Effect of pH on percentage recoveries of paraben extraction using CPE-DC193C-βCD-IL method.

The effect of sample pH on the recoveries of paraben was optimized over the range of 2–12. The results are exhibited in Figure 1. It is found that the CPE-DC193C-βCD-IL method shows that the extraction performance reached a better level at pH 9 for all parabens studied. As can be seen from Figure 1, the recoveries of parabens gradually increased until they reached the maximum value at pH 9 and gradually decreased after that. The results prove that the pH plays an important role in determining the optimum condition of paraben extraction. At a lower pH where the paraben is in protonated form, the extraction recovery of paraben in this form is low due to the repulsion between protonated parabens and the positive charge of βCD-IL [36].

Meanwhile, at pH 4–6.5, parabens exist mainly in neutral form. There is slight increase in the extraction recovery of paraben in this region as paraben loses its net positive charge due to the hydroxyl group becoming deprotonated. At pH values greater than 7 until pH 9, parabens exist mostly in a negatively charged form because the hydroxyl group is now fully deprotonated. In this region, the percentage recovery is increased dramatically and the maximum percentage recovery is achieved at pH 9. Thus, when parabens start to be deprotonated, the hydrogen bonding, electrostatic attraction and deprotonated interaction could be the main interaction between the positively charged βCD-IL with parabens, as discussed in Section 2.6 in detail.

### 2.2. Water Content in Surfactant-Rich Phase

We aim to get the lowest amount of water in the surfactant-rich phase when conducting the experiment in CPE-DC193C and CPE-DC193C-βCD-IL because the lower amount of water in the surfactant-rich phase results in the higher concentration of analyte that will be detected in the analysis. Thus, higher percentage recovery will be obtained. In addition, a good method was also produced as well as developed.

**Figure 2.**
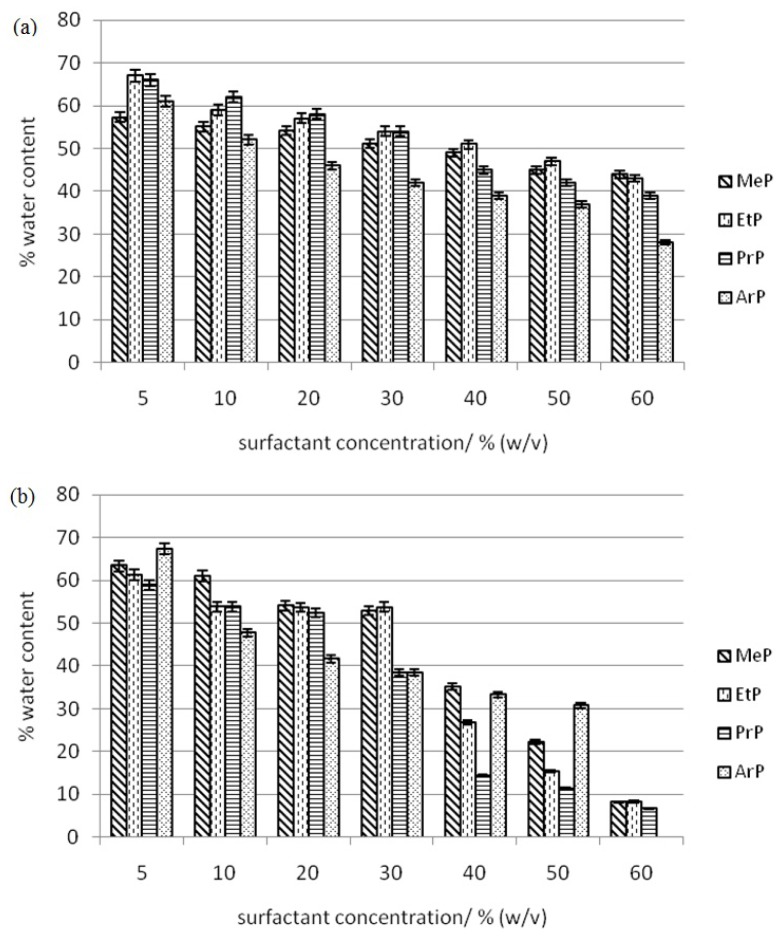
Water content in surfactant-rich of (a) CPE-DC193C and (b) CPE-DC193C-βCD-IL methods.

Based on Figure 2, ArP in the CPE-DC193C-βCD-IL method contains the highest amount of water at the beginning of experiments with almost 68% (*w*/*v*) of water content in the surfactant-rich phase at 5% (*w*/*v*) of surfactant concentration. This percentage was rapidly reduced to 0% (*w*/*v*) of water content at 60% (*w*/*v*) of surfactant concentration. A total loss of 68% was measured when the surfactant concentration was increased from 5% (*w*/*v*) to 60% (*w*/*v*) using the CPE-DC193C-βCD-IL method. This is considered the highest loss of water content when compared with the CPE-DC193C method. The overall loss of water content in the CPE-DC193C-βCD-IL method for methyl paraben (MeP) is 55%, followed by the ethyl paraben (EtP) and propyl paraben (PrP) with 52% respectively. This result clearly shows that the CPE-DC193C-βCD-IL method had produced the highest loss of water in the surfactant-rich phase.

CPE-DC193C shows the water content decreasing from almost 61% (*w*/*v*) to 28% (*w*/*v*) for benzyl paraben (ArP), when the surfactant concentration was increased. The highest value loss of water content was ArP with 33%, followed by PrP with 27%, EtP 24% and lastly MeP 13%. All analyte shows a decreasing trend in water content when the surfactant concentration is increased. This is because the solute–solute interaction between the surfactant itself has a greater contribution than the solute–water interaction when the amount of hydrophilic surfactant is getting higher in the solution. Therefore, the molecule of surfactant would prefer to interact with the same surfactant rather than interact with the water molecules, resulting in less amount of water detectable in the surfactant-rich phase. This phenomenon is also described by Sadeghi *et al.* [37]. They study the interaction between polymer surfactant polyethylene glycol with the salt or water. They conclude that solute–solute interaction between the studied polymer surfactant is greater than the interaction between surfactant with salt or water.

The method using βCD-IL shows the highest loss of water compared with CPE-DC193C because βCD-IL forms complex conformations with the surfactant, and the paraben in the surfactant-rich phase was thought to be present in the formation of micelles during the cloud point process. Thus, the spaces that remained for the water inside or among the micelles were efficiently compressed. Therefore, the amount of water in the surfactant-rich phase in CPE-DC193C-βCD-IL is small.

### 2.3. Distribution Coefficient

Table 1 illustrates the distribution coefficient of parabens in the two phases after the phase separation was completed. The values of log *K*d corresponded to the four parabens in the CPE-DC193C-βCD-IL and CPE-DC193C-βCD methods, which shows increases in the order of MeP < EtP < PrP < ArP. This behavior can be explained by considering the interaction between the parabens’ solubilization, the aggregation of DC 193C micelles, and the structure of the βCD-IL.

Both methods show the increases in the distribution coefficient when the hydrophobicity of the paraben increased. It can be explained clearly that the longer the chain, the higher the hydrophobicity. MeP shows the lowest value of log *K*d for both methods because this molecule has a shorter chain compared with the chains of the EtP, PrP and ArP. ArP shows the highest value of log *K*d because its structure is present in the aromatic group; thus, the hydrophobicity is the highest compared with other parabens studied. CPE-DC193C-βCD-IL shows high values of log *K*d compared with CPE-DC193C because of the presence of βCD in the complex. β-CD has the ability to form inclusion complex with the paraben because β-CD has the hydroxyl groups at the outer surface of the molecule, with the primary hydroxyls at the narrow side and secondary hydroxyls at the wider side, thereby making β-CD water soluble while simultaneously generating an inner cavity that is relatively hydrophobic.

β-CD can therefore either partially or entirely accommodate molecules of suitable size that are hydrophobic. In this situation, parabens form an inclusion complex with the inner part β-CD. Thus, the capability of βCD-IL as a modifier to extract a paraben from the aqueous phase into the surfactant-rich phase is higher. Therefore, the higher percentage recovery of parabens can be detected at the surfactant-rich phase when the phase separations are successfully formed. ArP shows the highest value of log *K*d because ArP is easily adsorbed into the cavity of β-CD that is relatively hydrophobic. ArP is the least polar and least soluble in water; ArP can therefore easily adsorb into the cavity of β-CD [23]. These findings were further correlated with the NMR result where it proves that ArP has been accommodating into the β-CD cavity, as will be discussed in detail in Section 2.6.

### 2.4. Phase Volume Ratio and Preconcentration Factor

**Figure 3.**
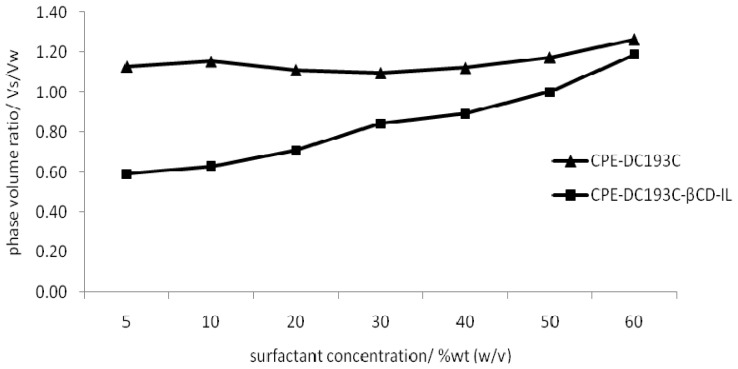
Comparison of phase volume ratio and preconcentration factor of MeP using CPE-DC193C and CPE-DC193C-βCD-IL method.

This study was conducted to investigate the effect of the phase volume ratio on the preconcentration factor. Figure 3 shows the phase volume ratio for CPE-DC193C-βCD-IL method and CPE-DC193C method corresponding to the surfactant concentrations. The lower phase volume ratio will produce a good result because it will reduce the final surfactant-rich phase, resulting in a higher preconcentration factor. Concomitantly, the higher recoveries of paraben extraction will be produced. The CPE-DC193C-βCD-IL method offers an obviously low phase volume ratio compared with the CPE-DC193C method, with the value of phase volume ratio 0.84 at 30% (*w*/*v*) surfactant concentration. The CPE-DC193C-βCD-IL method shows a marked increase on the phase volume ratio when the surfactant concentration is increased, at the same time proposing that the phase volume ratio of CPE-DC193C-βCD-IL is influenced by the surfactant concentration. Meanwhile, the CPE-DC193C method shows a constant value of the phase volume ratio when the surfactant concentration is increased. These results show that βCD-IL makes clear the phase separation and the surfactant-rich phase and aqueous phase form easily, thereby giving the concentration of the surfactant significant influence over the phase volume ratio of the CPE-DC193C-βCD-IL method.

From the CPE-DC193C-βCD-IL method experiment, we have obtained that at 30% (*w*/*v*), the surfactant concentration produced a sufficient volume of surfactant-rich phase. This amount is adequate for analysis using HPLC/UV. If more than 30% (*w*/*v*) surfactant concentration is used, the volume of surfactant-rich phase is insufficient for analysis using HPLC/UV, hence it does not produce a higher percentage of paraben recoveries. This is because at higher concentrations of surfactant, the viscosities of the surfactant are also higher, thus producing a small volume of surfactant-rich phase. This small volume of surfactant-rich phase is not sufficient to run the sample in HPLC/UV. In addition, the phase separation is also difficult to be observed. If less than 30% (*w*/*v*) surfactant concentration is used, the volume of surfactant-rich is sufficient for HPLC preparation, although it does not produce a higher percentage of recoveries. We decided to choose the phase volume ratio of 0.84 at 30% (*w*/*v*) surfactant concentration by considering the sufficient volume of surfactant rich phase and surfactant concentration that can produce a higher preconcentration factor. The sustained low phase volume ratio is highly advantageous in maintaining high paraben concentration in the surfactant rich phase, especially in the case of a high surfactant concentration, which would be proven in the preconcentration factor and distribution coefficient [38].

Supplemental data (Figure S1) shows the preconcentration factor of parabens using the CPE-DC193C-βCD-IL method and CPE-DC193C method. Both methods show an increase in the preconcentration factor with the increase on the hydrophobicity of the analyte. The developed method of CPE-DC193C-βCD-IL shows the highest value of preconcentration factor compared with the CPE-DC193C method. The values of the preconcentration factor of CPE-DC193C-βCD-IL for MeP, EtP, PrP and ArP are 76, 89, 97 and 110, respectively. Meanwhile, the values of the preconcentration factor of CPE for MeP, EtP, PrP and ArP are 57, 62, 79 and 85, respectively. The high values of the preconcentration factor for CPE-DC193C-βCD-IL method are due to the hydrophobicity of analyte that increased from MeP to ArP, resulting in ArP being the least polar and least soluble in water. Therefore, ArP can easily adsorb into the cavity of βCD [23]. Thus, a higher concentration of ArP will be detected in the surfactant-rich phase and will reduce the final surfactant-rich phase volume of the CPE-DC193C-βCD-IL process efficiently. Since a higher preconcentration factor is attributed to the smaller phase volume of the surfactant-rich phase, the volume of sample injected in the HPLC system was highly concentrated in paraben and produced good results. Therefore, we can conclude that the distribution of parabens in the surfactant-rich phase for CPE-DC193C-βCD-IL method depends on the hydrophobicity of parabens.

### 2.5. Method Validation

Based on the method development described above, the performances of the CPE-DC193C-βCD-IL method were tested by using environment water samples. The relative standard deviations, coefficient of determination, and limits of detection are shown in Table 2. Linear ranges of parabens for CPE-DC193C-βCD-IL method and CPE-DC193C method were 0.010–0.10 and 0.10–1.0 μg mL−1 respectively. Relative standard deviations correspond to the repeatability at concentration ranges from 0.010 to 0.10 μg mL−1. It shows that the developed method, namely the CPE-DC193C-βCD-IL method, gives a better performance in terms of the precision and lower limit of the detection of parabens.

The developed method was applied to the determination of the recoveries of parabens from a variety of water samples such as river, treated water, sea and tap water. The results are summarized in Table 3. The percentage extraction recovery of paraben, %*R* can be characterized as the percentage of paraben extracted from bulk solution into the surfactant-rich phase:

(1) $$\%R=\frac{C_{S}V_{S}}{C_{O}V_{t}}\times 100\%$$

where *Cs* (mg/L) is the paraben concentration in the surfactant-rich phase after phase separation and *C*o (mg/L) is the initial paraben concentration in the bulk solution before phase separation. While *V*s (mL) is the volume of the surfactant-rich phase and *V*t is the total volume of the solution [39]. The spiked paraben concentration in the real samples for the CPE-DC193C-βCD-IL and CPE-DC193C methods is 0.01 and 0.1 μg mL−1, respectively. Recovery rates using CPE-DC193C-βCD-IL were between 91.2% and 100% with relative standard deviations (RSD) of less than 1%. These results are comparable with the CPE-DC193C method. The recovery rate of the CPE-DC193C method was between 71.2% and 97.7%. In the CPE-DC193C method, it is reported that the lowest percentage of recovery was obtained for the sea water sample with the recovery range 72.1%–87.5%. This is probably due to the electrolyte factor (salt concentration) from sea water interrupting the CPE-DC193C method. The CPE-DC193C-βCD-IL method has successfully improved the percentage recoveries for paraben spiked in sea water samples with the value of 90.5%–93.7%. This is because the CPE-DC193C-βCD-IL method improves the selectivity of the paraben to form an inclusion complex in the inner cavity of the βCD-IL. The following results show that the method developed has a good recovery for the determination of the parabens from aqueous solution. These results have shown that the method developed is feasible to be used for monitoring paraben compounds in environmental water samples. The chromatograms of standard parabens and spiked paraben in water samples are illustrated in Figure S2a,b.

### 2.6. Extraction Behavior of ArP and DC193C with βCD-IL

**Figure 4.**
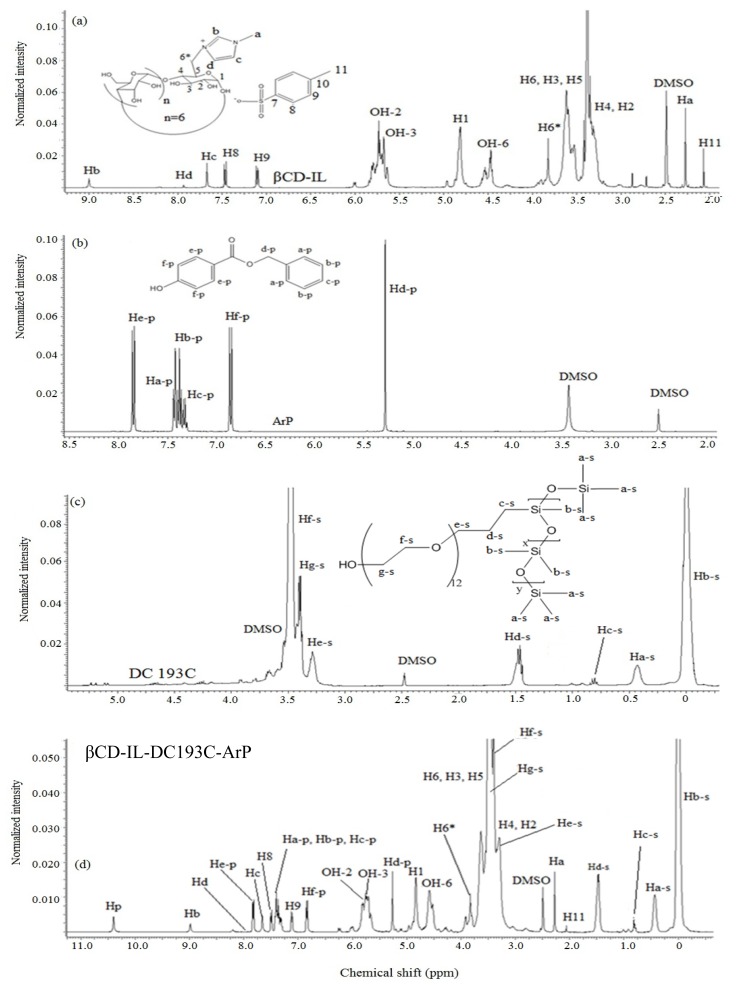
1H NMR spectrum of (a) βCD-IL; (b) ArP; (c) DC193C; and (d) βCD-IL-DC193C-ArP.

The analysis of the inclusion complex between modified βCD (βCD-IL), ArP, and the surfactant (DC193C) is very crucial in this work since the cavity of βCD was maintained during the extraction process. Furthermore, the findings supported that the inclusion complex formation is one of the main interactions that take place between βCD-IL, ArP, and DC193C in the extraction process. In order to evaluate the geometry of inclusion formation of βCD-IL, ArP and DC193C, 1H NMR (Figure 4) and 2D NOESY measurements (Refer to Figure S3) (DMSO-D6, 25 °C, 600 MHz) were performed on an AVN600 spectrometer. The obvious upfield shifts of the protons on the inner cavity of βCD-IL (H3, 3.5483 ppm and H5, 3.4848 ppm) were observed. This change indicates that the DC193C or ArP has been entered deeply into the cavity of CD. Based on the results obtained (Table 4), the protons of DC193C and ArP were found to be shifted upon the formation of inclusion complex (βCD-IL-DC193C-ArP). Meanwhile, H5 proton of βCD-IL changes from doublet to singlet upon the formation of inclusion complex as shown in Figure 4. The presence of 1H signals belonging to both βCD-IL, ArP and DC193C molecules could be observed in 1H NMR spectrum of βCD-IL-DC193C-ArP which strongly suggests that the new inclusion complex has been formed. Since in this study there are two guest compounds (DC193C and ArP), it is necessary to investigate further with 2D NMR in order to predict which one enters into the cavity of CD. There are a few NMR techniques that can provide supporting evidence for specific structures in cyclodextrin complexes. 2D-NOESY and 2D-ROESY experiments give rise to cross peaks between dipolar coupled spins [40,41], in order to indicate the close proximity between atoms in the two components of the complex. In addition, 2D NOESY and 2D ROESY experiments provide an upper limit (*ca.* 5 Å) on the distance between protons that produce cross peaks under favorable conditions.

The formation of an inclusion complex was further proven by the 2D-NOESY analysis (Refer to Figure S3) since 2D NMR is a powerful tool for investigating intermolecular interactions and to gain more information on the conformation of the inclusion complex [42]. The cross peaks in the spectra, indicated in SD3 originate from the interaction of the protons of DC193C, ArP and βCD-IL. The cross peaks of βCD-IL (3.5–3.6 ppm, H-3, H-5) and DC193C (Ha-s, 0.4314 ppm, Hb-s, 0.0001 ppm, Hd-s, 1.4795 ppm) demonstrate strong intensity. Hence, from the 2D NOESY spectra we can conclude that DC193C has been accommodated in the βCD cavity and may be within less than 5 Å apart from H3 and H5 of CD. Apart from that, 2D NOESY also shows interactions between βCD-IL and ArP. The cross peak (Hd-p, 5.2623 ppm; Hf-p, 6.8256 ppm) shows an interaction with βCD-IL (3.5–3.6 ppm, H-3, H-5) and it further supports that ArP has been accommodated in the cavity of CD.

**Figure 5.**
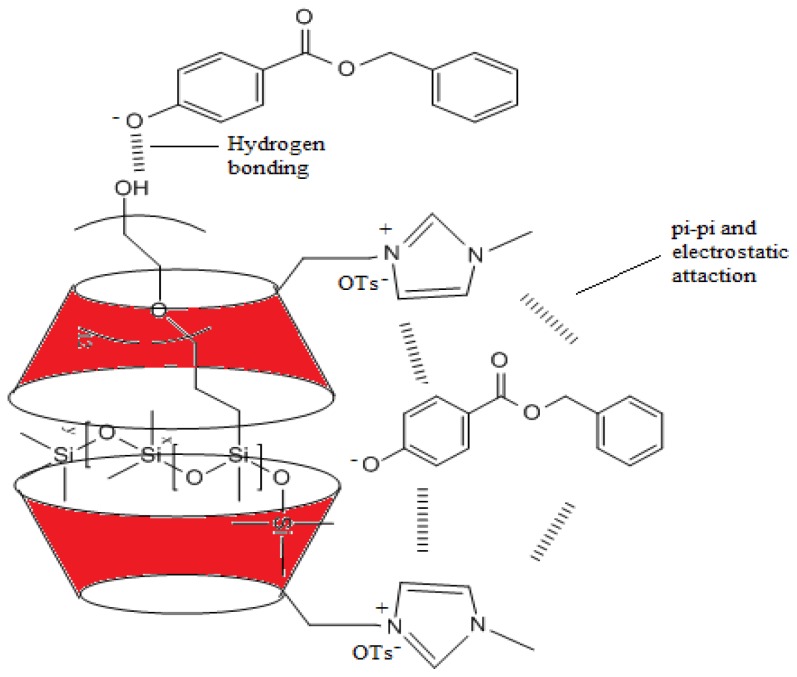
Schematic illustration of the pH-dependent complexation of ArP and DC193C with βCD-IL.

**Figure 6.**
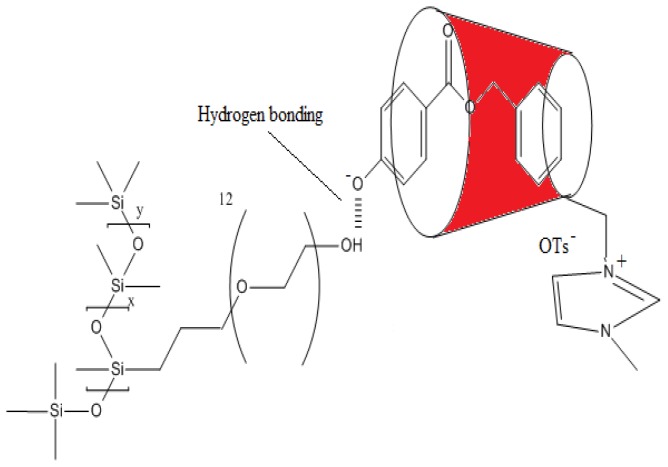
Schematic illustration of the pH-dependent complexation of ArP and DC193C with βCD-IL.

Apart from that, the cross peak around 6–8 ppm belonging to βCD-IL and ArP shows that there is an interaction between the imidazolium ring and ArP (Refer to Figure S3); this could be due to the π–π interaction and electrostatic attraction. Hence, the possible formations of the inclusion complex structure of the pH-dependent complexation of ArP and DC193C with βCD-IL are shown in Figures 5 and 6 and have been proposed by taking account of the hydrogen bonding between DC193C and deprotonated ArP, π–π interaction, electrostatic attraction between the imidazolium ring and ArP, as well as the inclusion complexes between βCD-IL with DC193C and βCD-IL with ArP.

## 3. Experimental Section

### 3.1. Reagents and Standards

Silicone–ethyleneoxide copolymer (DC 193C) was manufactured by Dow Corning (Shanghai, China) and supplied by Dow Corning Malaysia (Refer to Figure S4). Unfortunately, no information was available on the detailed molecular structure, where the values of *x*, *y* and molecular weight of these compounds were provided by the manufacturer. Methylparaben (MeP), ethylparaben (EtP), propylparaben (PrP) and benzylparaben (ArP) were purchased from Sigma–Aldrich (Buches SG, Switzerland). Acetonitrile (HPLC grade) was purchased from Merck (New York, NY, USA) and deionized water used in mobile phase was of conductivity 18 MΩ cm. Sodium sulfate (Na2SO4) was obtained from Merck (New York, NY, USA). Stock solutions of parabens at a concentration of 1000 mg/L were prepared in acetonitrile. Working standard solutions were prepared by step-wise diluting with deionized water of stock solutions. The pH of the solution samples was adjusted with diluted hydrochloric acid or diluted sodium hydroxide solutions. βCD is commercially available and was purchased from Acros (Geel, Belgium). 1-Methylimidazole was supplied from Sigma–Aldrich (Buches SG, Switzerland). *N*,*N*-Dimethylformamide (DMF) and hexane anhydrous were purchased form Merck (New York, NY, USA). *p*-Toluene sulfonic anhydride was prepared according to a literature procedure [43], and was used without further purification.

### 3.2. Instrumentation

The separation and quantification of the tested parabens were carried out on the Shimadzu HPLC system. The system consists of a pump, degasser, auto injector, column oven, ultraviolet detector, guard column, Chromolith C18 column (100 mm × 4.6 mm, Merck, New York, NY, USA). HPLC gradient conditions were used to separate the analytes using acetonitrile and deionized water, flow rate of 0.7 mL/min and detection at 254 nm. The gradient elution was performed as follows: 30% acetonitrile (0–2 min), ramped to 40% acetonitrile (3–5 min) and then ramped to 30% acetonitrile (5–8 min).

### 3.3. Synthesis β-Cyclodextrin Functionalized Ionic Liquid (βCD-IL) (1)

βCD-IL was prepared by reacting O-p-toluenesulfonyl-β-cyclodextrin (β-CDOTs) with 1-methylimidazole (MIM). β-CDOTs was prepared according to Zhong *et al.* [43]. Since tosyl is a good leaving group, imidazole can easily undergo nucleophilic substitution. The reaction was carried out in DMF solvent since β-CDOTs and MIM form a homogeneous solution. The preparation of mono-functionalized βCD with MIM (βCD-IL) was done according to the following procedure [32], as shown in Figure S5: Freshly dried CDOTs (1.00 g, 78 mmol) and appropriate amount of BIM (10 mole equivalent) in excess amount were dissolved in anhydrous DMF (40 mL) and the solution was stirred at 90 °C in an inert atmosphere. After two days, the resultant solution was cooled to room temperature and slowly added into acetone. Then the mixture was stirred for 30 min and thereafter filtered and washed in excess acetone. The obtained product was recrystallized three times from hot water to get the final product, 1 as a white precipitate. 1H NMR spectrum of βCD and βCD-OTs are shown in Figure S6. Figure 4a shows ^1^H NMR spectrum of compound **1**, βCD-IL in d_6_-DMSO solvent. The formed product was soluble in water and several organic solvents (DMF, DMS0 and ethanol). New peak was observed in proton (H6*, 3.86 ppm) and carbon signal (C6*, 49.8 ppm), belonged to a substituted CD.

IR/KBr, cm^−1^ 3301 (OH), 2919 (C–H), 1650 (C=C), 1152 (C–N). ^1^H NMR/ppm, DMSO-D_6_ Hb (9.0, s), Hd (7.93, s), Hc (7.67, s), Ha (2.27,s), H8 (7.47, d), H9 (7.11, d), OH2–OH3 (5.5–6.0, m), H1 (4,81, s), OH6 (4.4–4.6, m), H6* (3.86, s), H3, H5, H6 (3.5–3.6, m), H2–H4 (3.3–3.4, m), H11 (2.07, s). ^13^C NMR/PPM, DMSO D_6_ Cb (137), Cd (123.4), Cc (123), Ca (30.87), C7 (145.4), C10 (137.7), C9 (128.05), C8 (125.4), C1 (101.64), C4 (81.5), C2 (73.3), C3 (72.6), C5 (72.1), C6 (59.9), C6* (49.8), C11 (21.9). CHNS (%) C (37.4), H (7.51), S (0.54), N (1.13). Percentage yield (90%).

### 3.4. Preparation for Cloud Point Extraction (CPE)

In the CPE-DC193C-βCD-IL experiments, a desired aqueous solution was obtained by blending 1.0 mL 30% (*w*/*v*) surfactant aqueous solution, 1.0 mL of βCD-IL (10 ppm), 1 mL of stock solution of parabens, 0.5 mL of sodium sulfate solution using ultrasonicator for 5 min. pH was adjusted using diluted acid or diluted alkaline solution. The cloud point was determined by measuring the temperature of the solution when the solution became turbid after being heated in a thermostatic waterbath at 30 °C and then cooled to room temperature. Subsequently, the separation of the phases was achieved by centrifugation for 10 min at 4000 rpm or otherwise kept overnight to ensure the separation between the surfactant’s rich phase and water was achieved. Then, the volumes of surfactant-rich phase and water were measured. In the CPE-DC193C-βCD-IL experiments, the same method as in the CPE-DC193C-βCD-IL experiments was applied without the addition of βCD-IL.

### 3.5. Analysis of Water Samples

Tap water samples were collected from the laboratory. River water samples were collected from Bahau, Negeri Sembilan (geographical coordinate 3°1′44″N 102°22′1″E, Malaysia), while treated water samples were collected from a wastewater treatment plant in Kuala Lumpur (geographical coordinate 3°7′25″N 101°39′12″E, Malaysia) and sea water samples were collected from Perak (geographical coordinate 4°13′0″N 100°34′0″E, Malaysia). All water samples were filtered using a 0.45 μm nylon membrane filter to remove suspended particulate matters and then stored at 4 °C in the dark. Then 1.0 mL of water samples was added to the CPE-DC193C-βCD-IL preparation (as mentioned above in Section 2.2) and analyzed using HPLC-UV.

### 3.6. Preparation and Characterization of Inclusion Complex of βCD-IL, Surfactant DC193C and Benzyl Paraben

The inclusion complex of βCD-IL with non-ionic surfactant, DC 193C and benzyl paraben (ArP) was prepared using the conventional kneading method in basic condition [44]. Equimolar amounts of βCD-IL, surfactant DC193C and ArP, were kneaded with mortar and pestle in minimal ethanol to form a homogeneous paste. The complex was kneaded for approximately 30 min and dried to constant mass. After drying, a white powder (βCD-IL-surfactant DC193C-ArP complex) was obtained. ^1^H NMR and 2D NOESY spectra were recorded on AVN 600 MHz and DMSO-d_6_ was used as solvent.

## 4. Conclusions

This study demonstrates that the CPE-DC193C-βCD-IL consisting of mono-6-deoxy-6-(3-methylimidazolium)-β-cyclodextrin as a modifier serves as an excellent strategy for the extraction of parabens from various water samples. CPE-DC193C-βCD-IL with βCD-IL as modifier give higher distribution coefficient, good preconcentration factor and higher recovery compared with the CPE-DC193C. The addition of βCD-IL as a modifier increases the ability to form phase separation between the ionic liquid-rich and aqueous phases. The addition of βCD-IL also proves that the percentage of water content in the surfactant-rich phase is lower than that in the CPE-DC193C method. The distribution coefficient of the four parabens studied will be increased gradually when βCD-IL is added. By taking into account the CPE-DC193C-βCD-IL and CPE-DC193C, using βCD-IL is economically viable as it improves the performance of the CPE-DC193C method dramatically. Moreover, this chemical is inexpensive and is not toxic to our environment. Finally, the inclusion complex formation, hydrogen bonding and π–π interaction between the βCD-IL, ArP and DC193C has been proven using ^1^H NMR and 2D NOESY spectroscopy.

## Supplementary Information



## Figures and Tables

**Table 1.** Comparison of distribution coefficients of the studied paraben on CPE-DC193C and CPE-DC193C-βCD-IL.

Log *K*_d_	CPE-DC193C	CPE-DC193C-βCD-IL
MeP	1	3.2
EtP	2.6	3.6
PrP	3.2	4.6
ArP	3.8	4.9

**Table 2.** Relative standard deviations, coefficient of determination, and limits of detection of the method developed on the determination of parabens from aqueous solution.

Analyte	Precison, coefficient of determination and limit of detection	CPE-DC193C	CPE-DC193C-βCD-IL
**MeP**	RSD (% *n* = 3)	0.17	0.28
	Coefficient of determination, *R*^2^	0.998	0.990
	LOD (μg mL^−1^)	0.29	0.038

**EtP**	RSD (% *n* = 3)	0.45	0.86
	Coefficient of determination, *R*^2^	0.991	0.991
	LOD (μg mL^−1^)	0.23	0.026

**PrP**	RSD (% *n* = 3)	0.66	0.36
	Coefficient of determination, *R*^2^	0.986	0.993
	LOD (μg mL^−1^)	0.21	0.016

**ArP**	RSD (% *n* = 3)	0.47	0.13
	Coefficient of determination, *R*^2^	0.993	0.991
	LOD (μg mL^−1^)	0.14	0.013

**Table 3.** Recovery of parabens in spiked water samples in the method developed.

Method	Analyte	River water recovery, % RSD, %	Tap water recovery, % RSD, %	Treated water recovery, % RSD, %	Sea water recovery, % RSD, %
CPE-DC193C	MeP	96.2 (0.47)	83.8 (0.59)	85.9(0.2)	72.1 (0.62)
	EtP	93.8 (0.15)	96.3 (0.76)	87.7(0.30)	71.2 (0.55)
	PrP	97.7 (0.63)	93.3 (0.26)	94.3(0.40)	87.9 (0.23)
	ArP	89.5 (0.29)	80.6 (0.67)	85.6(0.34)	85.8 (0.40)

CPE-DC193C-DC193C-βCD-IL	MeP	97.5 (0.35)	92.3 (0.26)	97.8 (0.22)	96.2 (0.32)
	EtP	98.9 (0.80)	94.9 (0.83)	92.9 (0.96)	91.2 (0.72)
	PrP	97.4 (0.15)	97.8 (0.42)	96.1 (0.48)	93.2 (0.64)
	ArP	97.6 (0.63)	95.5(0.34)	100.0 (0.49)	98.2 (0.68)

**Table 4.** ^1^H NMR chemical shift (§) of βCD-IL, ArP, DC193C and βCD-IL-ArP-DC193C.

Proton	βCD-IL	ArP	βCD-IL-ArP-DC193C	
	
	§	§	§	Δ§
H1	4.8191		4.8315	+0.0124
H2	3.3119		3.3445	+0.0326
H3	3.5987		3.5483	**−0.0504**
H4	3.3656		3.3945	+0.0289
H5	3.5517		3.4848	**−0.0669**
H6	3.6176		3.6331	+0.0155
H8	7.4545		7.4847	+0.0302
H9	7.0939		7.1057	+0.0118
H11	2.0677		2.0594	−0.0083
Ha	2.2752		2.2760	+0.0008
Hb	9.0063		8.9827	−0.0236
Hc	7.6730		7.6586	−0.0144
Hd	7.9300		7.9500	+0.0200

Ha-p		7.4200	7.4023	−0.0177
Hb-p		7.3770	7.3669	−0.0101
Hc-p		7.3370	7.3272	−0.0098
Hd-p		5.2780	5.2623	−0.0157
He-p		7.8530	7.8154	−**0.0376**
Hf-p		6.8730	6.8256	−**0.0474**

**Proton**		**DC193C**	**βCD-IL-ArP-DC193C**	
		
		**§**	**§**	**Δ§**

Ha-s		0.4320	0.4314	−0.0006
Hb-s		0.0305	0.0001	−**0.0304**
Hc-s		0.8262	0.7951	−0.0238
Hd-s		1.4401	1.4795	−**0.0384**
He-s		3.3780	3.2938	−**0.0842**
Hf-s		3.5257	3.4848	−**0.0414**
Hg-s		3.4037	3.3945	−0.0092
