# Supplementary material for: Cloud Point Extraction of Parabens Using Non-Ionic Surfactant with Cylodextrin Functionalized Ionic Liquid as a Modifier
Source: Int J Mol Sci. 2013 Dec 17;14(12):24531–48. doi: 10.3390/ijms141224531 (PMC3876126; doi:10.3390/ijms141224531)

Supplementary Information

Figure S1. Comparison of preconcentration factor of studied parabens using CPE-DC193C and CPE-DC193C-βCD-IL.

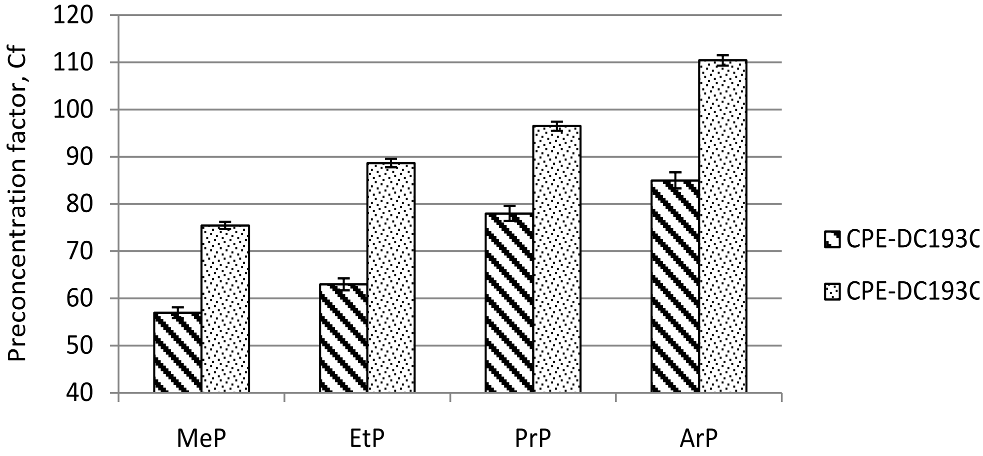

Figure S2. (**a**) Chromatogram of mixture of parabens; (**b**) Chromatogram of samples of sea water spiked with parabens. Amount of spiked at 400 ng/L of MeP, EtP, PrP and ArP. Cloud point conditions: 2.0 wt% (*w*/*v*) DC 193C. HPLC condition: Chromolith C18 column (100 mm × 4.6 mm). HPLC gradient conditions were used to separate the analytes using acetonitrile and deionized water, flow rate of 0.7 mL/min and detection at 254 nm. The gradient elution was performed as follows: 30% acetonitrile (0–2 min), ramped to 40% acetonitrile (3–5 min) and then ramped to 30% acetonitrile (5–8 min). A = methyl paraben, B = ethyl paraben, C = propyl paraben, D = benzyl paraben.

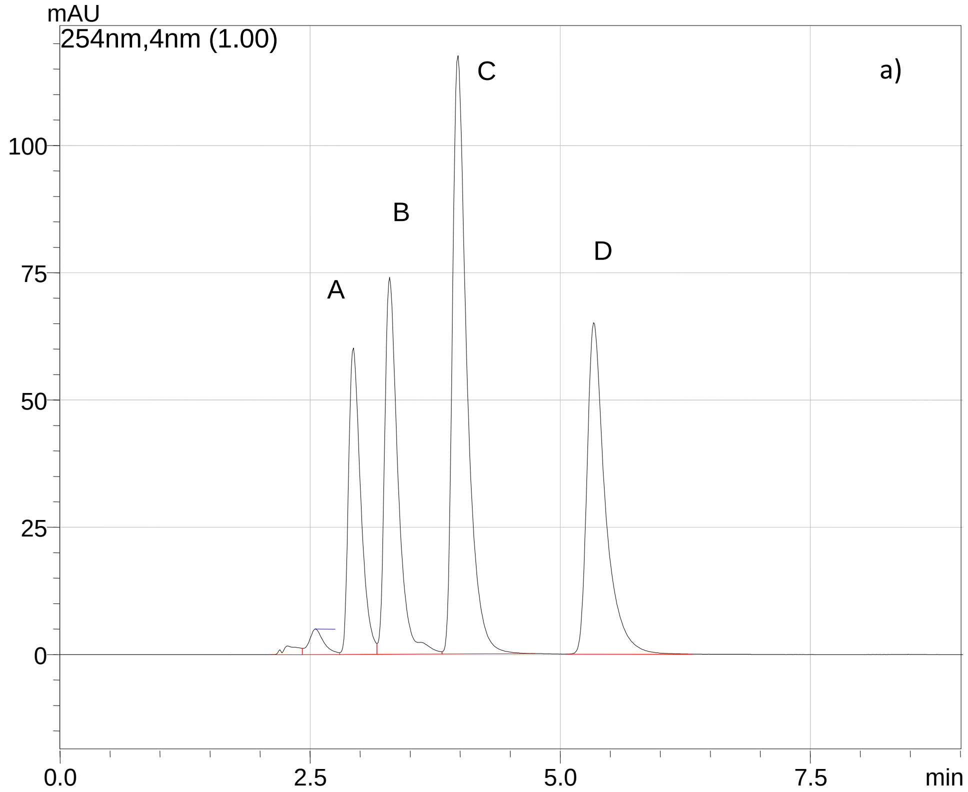

Figure S2. *Cont.*

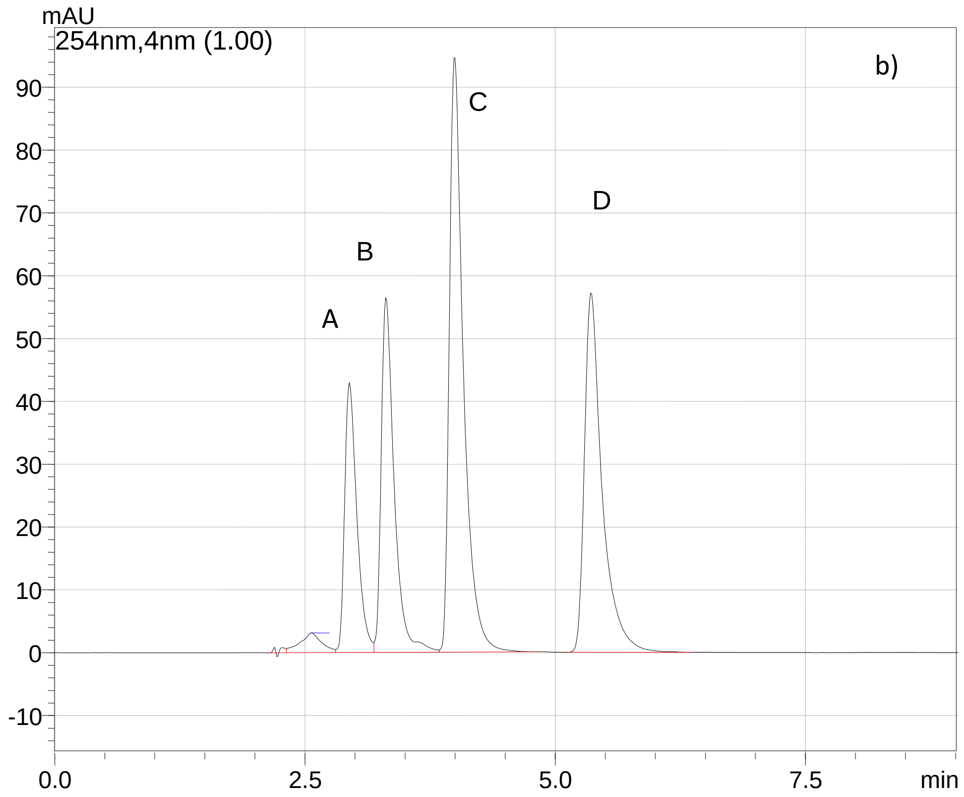

Figure S3. Two-dimensional NOESY spectrum of βCD-IL-DC193C-ArP complex in DMSO-D_6.

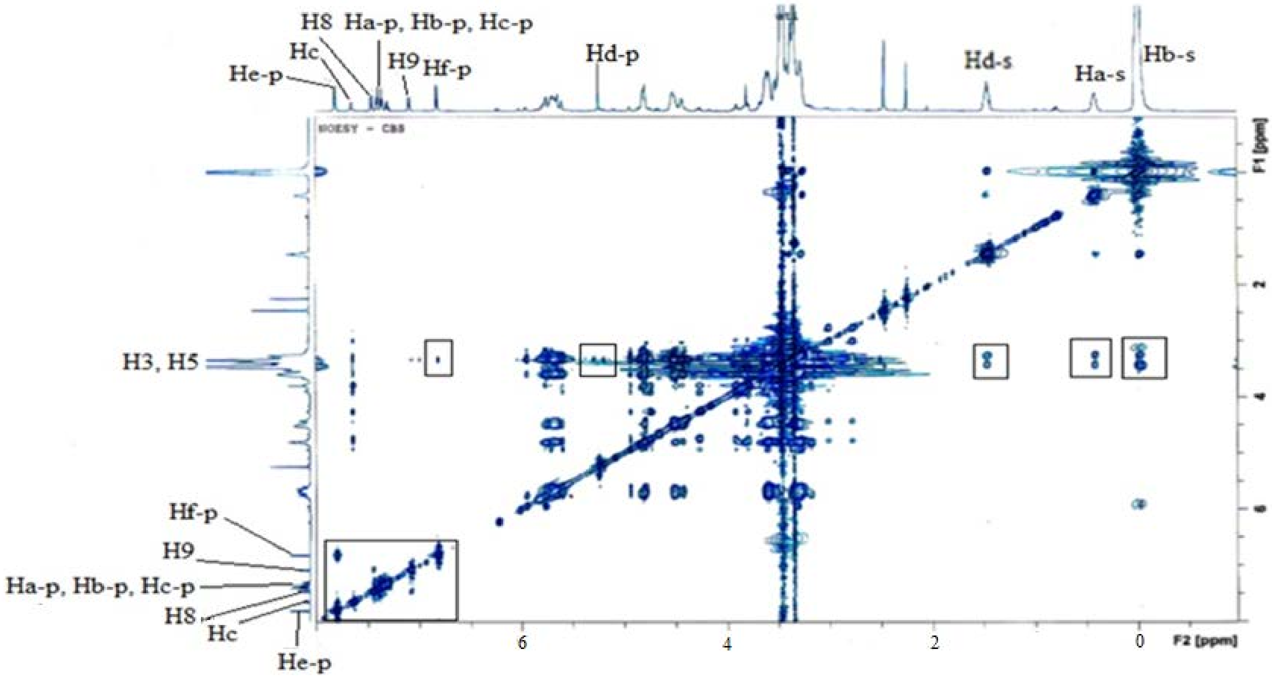

Figure S4. Structure of non-ionic surfactant DC193C.

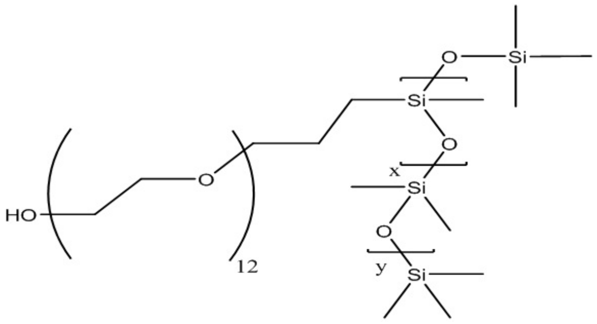

Figure S5. Preparation of βCD functionalized IL (βCD-IL).

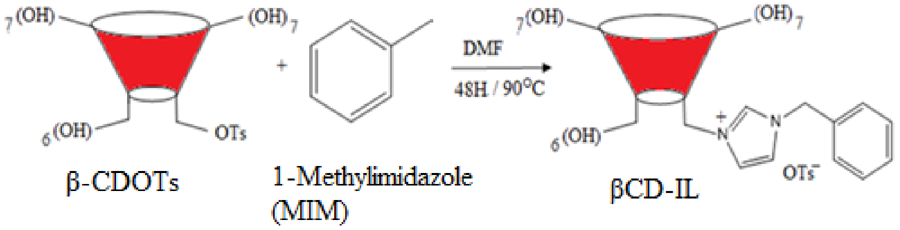

Figure S6. ^{1}H NMR spectrum of (**a**) βCD; and (**b**) βCD-OTs.

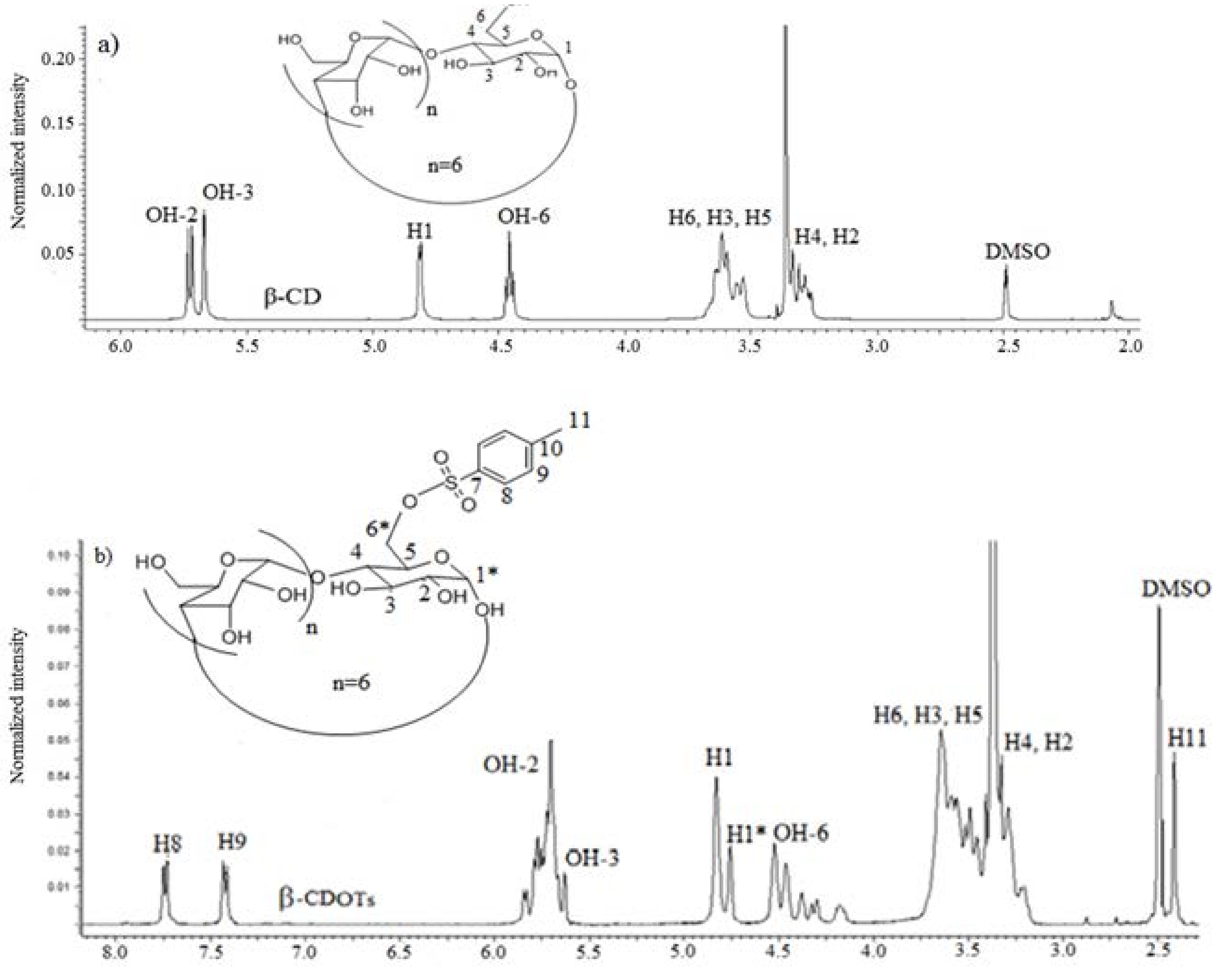

Supplement: Supplementary file 1 [file ijms-14-24531-s001.pdf]
